# Bioinformatics Accelerates the Major Tetrad: A Real Boost for the Pharmaceutical Industry

**DOI:** 10.3390/ijms22126184

**Published:** 2021-06-08

**Authors:** Tapan Behl, Ishnoor Kaur, Aayush Sehgal, Sukhbir Singh, Saurabh Bhatia, Ahmed Al-Harrasi, Gokhan Zengin, Elena Emilia Babes, Ciprian Brisc, Manuela Stoicescu, Mirela Marioara Toma, Cristian Sava, Simona Gabriela Bungau

**Affiliations:** 1 Department of Pharmacology, Chitkara College of Pharmacy, Chitkara University, Punjab 140401, India; ishnoorkaur7@gmail.com (I.K.); aayushsehgal00@gmail.com (A.S.); sukhbir.singh@chitkara.edu.in (S.S.); 2 Amity Institute of Pharmacy, Amity University, Gurugram 122413, India; sbsaurabhbhatia@gmail.com; 3 Natural & Medical Sciences Research Centre, University of Nizwa, Birkat Al Mauz, Nizwa 616, Oman; aharrasi@unizwa.edu.om; 4 Department of Biology, Faculty of Science, Selcuk University Campus, 42130 Konya, Turkey; biyologzengin@gmail.com; 5 Department of Medical Disciplines, Faculty of Medicine and Pharmacy, University of Oradea, 410073 Oradea, Romania; babes.emilia@gmail.com (E.E.B.); brisciprian@gmail.com (C.B.); manuela_stoicescu@yahoo.com (M.S.); cristian.sava2004@gmail.com (C.S.); 6 Department of Pharmacy, Faculty of Medicine and Pharmacy, University of Oradea, 410028 Oradea, Romania; mire.toma@yahoo.com; 7 Doctoral School of Biomedical Sciences, University of Oradea, 410087 Oradea, Romania

**Keywords:** bioinformatics, pharmacovigilance, microbiology, microRNA, COVID-19, public health

## Abstract

With advanced technology and its development, bioinformatics is one of the avant-garde fields that has managed to make amazing progress in the pharmaceutical–medical field by modeling the infrastructural dimensions of healthcare and integrating computing tools in drug innovation, facilitating prevention, detection/more accurate diagnosis, and treatment of disorders, while saving time and money. By association, bioinformatics and pharmacovigilance promoted both sample analyzes and interpretation of drug side effects, also focusing on drug discovery and development (DDD), in which systems biology, a personalized approach, and drug repositioning were considered together with translational medicine. The role of bioinformatics has been highlighted in DDD, proteomics, genetics, modeling, miRNA discovery and assessment, and clinical genome sequencing. The authors have collated significant data from the most known online databases and publishers, also narrowing the diversified applications, in order to target four major areas (tetrad): DDD, anti-microbial research, genomic sequencing, and miRNA research and its significance in the management of current pandemic context. Our analysis aims to provide optimal data in the field by stratification of the information related to the published data in key sectors and to capture the attention of researchers interested in bioinformatics, a field that has succeeded in advancing the healthcare paradigm by introducing developing techniques and multiple database platforms, addressed in the manuscript.

## 1. Introduction

Bioinformatics is a term that refers to the collection and evaluation of scientific data by employing computational techniques, integrating the biological information comprising of proteins, genes, cells, robotics, medical information, and ecosystems with technological mediums such as databases, software, tools, etc. [1,2,3]. Bioinformatics was completely established as a significant field by the 1990s, with an established role in the scientific paradigm [4]. Numerous economical bioinformatics tools have been established as a result of accelerating technological advancements [5]. The mRNA profiling and genomic sequencing techniques have transformed the disease detection and management approaches [3]. The conduct of clinical trials requires significant time and massive capital investment in introducing a drug into the market, thereby establishing a dire need for an economical approach to discover and develop drugs, followed by their overall assessment and evaluation [6].

The pharmacological therapies are available for only 30% of the diseases identified, as per the investigations [7], and many biological targets for numerous diseases are yet to be identified [8]. Bioinformatics integrates biostatistical aspects and computational techniques with biomedical sciences, like genetics, proteomics, epidemiology, and genomics [9]. Bioinformatics aims to enable data analysis, management, and interpretation from observational investigations and biological studies, therefore targeting implementation and development of databases, biological inference and interpretation, as well as data evaluation and mining, which is very well managed by the National Center for Biotechnology Information (NBCI), European Bioinformatics Institute (EBI) [9], Riken (Japanese National Research and Development Agencies), etc. 

Bioinformatics has been introduced in the pharmaceutical industry on the basis of two significant aspects, computational chemistry and molecular biology, where the latter is a specific and targeted approach, focusing on genetics and sequencing of data, which is a necessary biomedical tool, while the former emphasizes structural analysis more than biological applications, comprising crystallographic techniques, protein-structure determination, etc. [10]. Molecular biologists adopt a sequence-based approach to bioinformatics, whereas computational chemists employ bioinformatics in terms of protein structures [10]. In the pharmaceutical industry, bioinformatics has occupied a fundamental position by facilitating the management and organization of novel developments and sectors, such as pharmacovigilance, which provides a significant basis for drug development and research, and enables the identification, evaluation, prevention, and understanding of the adverse effects or reactions as a result of drug administration [11]. “Biomedical informatics” is defined by the American Medical Informatics as an interdisciplinary approach developed to evaluate and understand the utilization of information and data of biomedical importance, along with knowledge for scientific inquiry, decisive ability, and analytical thinking to solve problems, synergized by steps to enhance human health [12,13,14,15,16]. The techniques associated with bioinformatics have been found to benefit the objectives of the pharmaceutical and medical sector, due to the availability of large-scale datasets, such as methodologies of collecting useful safety information from newer resources, such as electronic health records [12]. Pharmacovigilance has been reported to be influenced by basic principles of bioinformatics, such as storage, decision making, computational skills, data generation, retrieval, use, communication, or sharing, which enable the production of a suitable framework to promote the organization of events associated with pharmacovigilance [13]. The healthcare system has reached the so-called “third wave” of digitalization, according to McKinsey, due to the application of innovative techniques, which are patient-centric, targeting the development of required services, which further propagates more intense innovations in pharmacovigilance and the healthcare sector.

Bioinformatics has been found to play a significant role in case processing in the pharmaceutical industry, using computational collection of data, followed by entry of the complete data, evaluation of expectedness and casualty, quality control (QC), narrative writing, and submission of reports, as well as storage and maintenance of data [14,15]. Additionally, information related to drug safety management, summary of clinical safety information, updated data related to global drug safety regulations, participation in meetings and training sessions of operational staff on issues related to drug safety, adverse drug reaction (ADR) reporting to the regulatory agencies, QC work of other staff, and taking over a task given by the medical director or the manager within the abilities of the drug safety associate are appropriately carried out [14,16].

The present review enlists the tools and databases based upon bioinformatics, in tabular form throughout the body, in order to simplify the applications for the readers, thereby maintaining the interest and relevance of the review throughout. It also throws light on the significance of technical approaches to the management of the current situation of coronavirus disease (COVID-19). Moreover, this review proceeds to attract the focus of global researchers towards the functional aspects of bioinformatic strategies and invokes the need to establish awareness about this field in the healthcare sector in order to accelerate the potential and rate of advancements to enable rapid diagnosis, screening, treatment, and prevention of disorders, along with better and reliable management of problems related to public health.

## 2. Methodology

**Figure 1:**
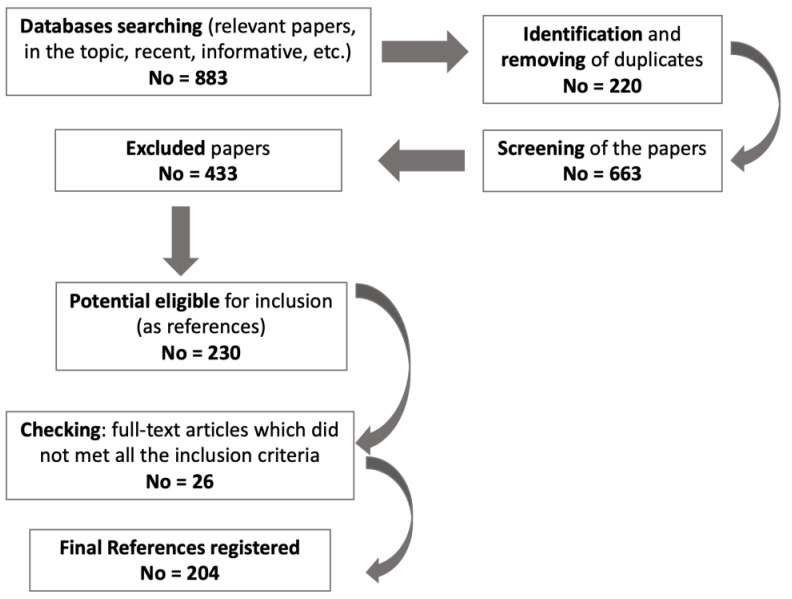
Flow chart presenting the methodology of published data selection.

The review has incorporated comprehensive data collected from multiple online platforms, publishers, and databases (i.e., Pub Med, MDPI, National Center for Biotechnology Information–NCBI, Google Scholar, Elsevier, Springer, Research Gate, etc.), after thorough research, associated with the role of bioinformatics in the healthcare sector. Competent selection criteria have been considered by selecting articles targeting the role of bioinformatics in the pharmaceutical and medical sectors in particular to provide a localized approach in the present era. The flow chart presented in Figure 1 describes the selection methodology of the articles.

The authors have referred to multiple articles from some specific journals in bioinformatics (Briefings in Bioinformatics; Network Modeling Analysis in Health Informatics and Bioinformatics; Proteins: Structure, Function, and Bioinformatics; Journal of Biomedical Informatics, Bioinformatics Database, etc.) with year of publication primarily ranging from 2010 to 2021; also, they aimed to design a thorough review, with an objective to expound the existing and future trends of bioinformatics tools and techniques in the pharmaceutical and medicinal sectors, beginning from their descriptive role in the pharmaceutical industry and pharmacovigilance, following which its applications in prime areas has been elaborated. The role of bioinformatics was segregated into four major areas, forming a tetrad in the healthcare sector as follows: drug discovery and development, anti-microbial research, genomic sequencing, and miRNA research and COVID-19 management.

## 3. Bioinformatics as an Asset of the Drug Discovery and Development (DDD) Paradigm

The selection of appropriate macromolecular targets is an important facet of the drug discovery process, which is performed after complete knowledge of the disease, which is followed by screen building and selection of lead compounds [10]. Medicinal chemistry approaches are then used for the optimization of leads to develop compounds that can be propagated to clinical trials. The first step of information gathering considerably employs computational methods, which comprise access to online literary databases, in-house collection of chemical structures, and data related to screening and biological assay [17]. In the laboratories, where the access to such tools is limited to just the specialists, bioinformatics approaches can catalyze the flow of these tools towards the bench scientists as well. Prior to the target identification, preliminary investigations are carried out, which comprise experimentation, study of biochemical processes, disease phenotypes, genome information, genetic linkage data, etc., using public domain software and other bioinformatics approaches [18]. A significant advantage is that these processes are dynamic and data collection is rapid, allowing the query to be repeated periodically to obtain complete and necessary information via systems referred to as alerting systems, which are moving forward into literature searching and bioinformatics, contributing to drug discovery [10]. Furthermore, efficient tools should be made available to facilitate the construction of computational models for significant processes by using selected and retrieved data from multiple sources [19]. Bioinformatics shortens the time taken by the preliminary stage of the drug discovery process by enabling browsing of large quantities of systemically evaluated genomic data on the desktop [10].

**Figure 2:**
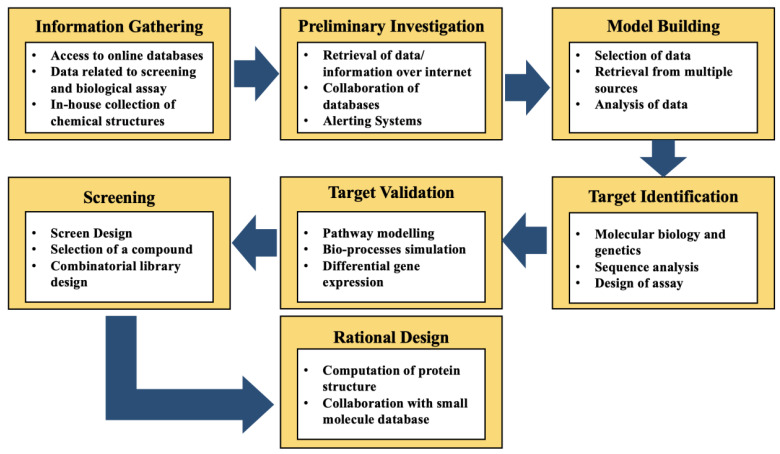
The multiple stages of drug discovery and the events targeted in each of the discussed steps.

Study of the mode of action of compounds, resulting from screens, is enabled by the identification of well-defined targets and genes associated with the disease that can be performed by sequence analysis and methods of computational molecular biology, alongside multiple databases of sequences and programs that employed comparison, analysis, and prediction of properties of sequences [20]. Moreover, once the targets and their functions are identified, it is necessary to exhibit their validation as targets. This is enabled by generating computational models associated with the biochemical pathways of the genes/targets in question, which can further make it possible to recognize the most suitable point for therapy [10]. Multiple gene expression techniques can further help to differentiate the pattern of gene expression profiles between healthy and diseased states, which can further be modeled to promote the effect of intervention at multiple points. The following stage promotes the use of a target, which is validated, in a high throughput screen (HTS) to facilitate the recognition of lead compounds by adapting either an existing assay or developing a new one, which requires significant molecular biology and other bioinformatics approaches [10]. Bioinformatics can be significantly employed to guide the chemists involved in the synthesis of combinatorial libraries, which focuses on the appropriate chemical properties of macromolecular targets, to promote the identification of small molecules with relevant activity from the screen. The structural information, available for the target in question, decides the contribution of bioinformatics to the process of rational designing, where the significant properties of the molecules should be evaluated to define their significance and potential in medicine. These properties include toxicity profile, selectivity, potency, and pharmacokinetic parameters [10]. Figure 2 represents the steps in the process of drug discovery and the events that follow.

The research process transits to the development phase when it is no longer possible to further improve or modify the molecule, where the evaluation of safety and efficacy parameters become a significant consideration, prior to becoming a medicine. Bioinformatics is also a pivotal tool in the development phase, in addition to the research, where it accelerates the understanding of the genetic profile of the individuals receiving the drug before its administration, which would make it necessary to genotype the subjects to be sure that they will benefit from the medicine [10].

### 3.1. Translational Bioinformatics and Systems Biology in DDD

The discovery and development of newer drugs with greater efficacy in complex disorders is a significant application of translational bioinformatics and systems biology [21]. Certain drawbacks are associated with conventional methods of DDD (post-market surveillance and clinical trials), such as the small size of samples, limited clinical data, and biased analysis [22]. Such challenges and issues can be resolved by progressive advancements in high-throughput analysis (HTP) and functional genomics by introducing better and more convenient techniques in the processes of drug designing [23]. A prospective and retrospective evaluation occurs as a result of applying computational and experimental methods in systems biology, facilitating the identification of more efficacious and personalized options in medications [23].

The complex network of protein–protein interactions is called the “interactome” [24], which can be elucidated by systems biology approaches, such as translational bioinformatics, and HTP technology of multiparametric data sets, in order to aid the development and targeting of more reliable drug candidates. Such techniques have exhibited useful applications in portraying the interactive motifs in disorders such as neuronal degeneration diseases [25]. Furthermore, the cellular alterations can be examined and described in detail at the cellular level using high content screen-based systems biology strategies [26], where the information obtained can be collated in proteomic and transcriptomic profiles, along with the relationship to phenotypic disorders and drug reactions.

Certain obstacles need to be overcome in translational bioinformatics in order to facilitate effective drug discovery, such as execution and compilation of multiple types of data from studies associated with systems biology, the interrelationship among complex attributes and entities, and the need for data mining with greater efficiency and more reliable decision support tools, as well as more comprehensive data designs [21]. From the “translational” perspective, the strategies of systems biology can be employed in the process of drug discovery by wrapping the network-depending designs, targeting molecular and cellular interactions, which may facilitate more rapid target validation and ameliorated attrition rates [27]. This system-based strategy may be useful in the discovery of multidrug therapies and combination of drugs [21].

Drug repositioning is a convenient tool for drug discovery that, with the identification of patient subgroups as well as novel combinations of conventional drug candidates, may enhance the effectiveness of therapy and abbreviate the zooming expenses in the healthcare system [21]. The drug repositioning approach can be supported by the translational bioinformatics approach to facilitate analysis of transcriptomic data and identification of the biological relationship between drug candidates and health disorders, which is a pivotal step towards personalized medicine [21]. Such systems-based designs may also be used in the treatment of cancer and cardiovascular disorders by targeting the network interactions in migration and proliferation of cells, as well as drug resistance [21].

Further, from a “bioinformatics” perspective, effective management of a massive number of drug interactions and combinations is a significant task. More reliable screening and decision-making for combination of drugs are enabled by integrated applications of HTP technologies, systems biology, and translational bioinformatics approaches [21]. Therefore, systems biology and bioinformatics techniques account for multilevel designing of functional genomics and signaling pathways [28]. Strategies, such as models based upon statistical association, would be applicable for recognizing biomarkers, depending upon the network and “interactome” signatures for optimum targets, further enabling computer-aided drug design (CADD) and poly-pharmacological approaches [29].

### 3.2. Discussion of Methods, Tools, and Databases Based on Translational Bioinformatics

Certain bioinformatics approaches and databases can be employed in translational studies associated with drug discovery and development, such as absorption of the drug, distribution profile, metabolic processes, excretion, and toxicity profile (ADMET) along with an integrated database for ADMET, as well as adverse effect predictive modeling (IDAAPM), which provides predictive designing, alongside evaluation and analysis of drug information provided by the Food and Drug Administration (FDA) [29]. The analysis of drug targets and discovery is found to be promoted by the drug-minded protein interaction database (DrumPID) [30]. Significant information related to structural inter-atomics is collected by CREDO to aid in the development of drugs [31]. Moreover, a protein–drug interaction database (PDID) is a database associated with an interactive relationship between proteins and drugs in the human proteome [32]. A manually and carefully selected database, the Orphan Nuclear Receptor Ligand Binding Database (ONRLDB), employs ligand moieties for orphan nuclear receptors for drug modeling [33]. A platform integrates genomic information with drug response data, known as the Mutations and Drug Portal (MDP) [34]. A Virtually Aligned Matched Molecular Pairs Including Receptor Environment (VAMMPIRE) database is associated with similar molecular pairs to aid in the development of drug models as well as optimization based upon structural elucidations [35].

Other databases also exist such as the cancer drug resistance (CancerDR) database (associated with cancer drug resistance), ChEMBL (drug model promoting large-scale bioactivity database), the metabolism and drug interaction database (associated with the evaluation of drug interactions), and PROMISCUOUS (concerned with drug repositioning based upon network) [36]. The treatment portfolio primarily focuses on the later stages of pathology with low predictive values and potential rates [37], but investigations associated with environmental and genomic interactions facilitate the development of more reliable preventive approaches. This process is aided by translational bioinformatics, which is collated with the clinical drug discovery and research data towards the development of a personalized approach to medicine [21]. The datasets for disease classification, discovery of biomarkers, drug targeting, network modeling, and drug repositioning can be effectively organized by translational bioinformatics. The disease models, based upon multiscale networks (possessing great predictive values), can be collated with genomic data related to clinical characteristics and other aspects [21]. The prime driving agents and biomarkers of the disease can be recognized by causal network interference models [37].

It is noteworthy that the information from proteomics and transcriptomics data, as well as HTP technologies, is effectively interpreted by data integration, which forms a potential aspect of computational drug designing and translational bioinformatics [38]. Furthermore, data warehouses, federated databases, as well as semantic technology would promote retrieval of data, discovery of novel drugs, and clinical screening of disease. Not only the biomedical aspects but also the translational bioinformatics approaches aid in overpowering the challenges associated with the pharmaceutical industry [39]. The methylated genes related to drug resistance associated with ovarian cancer were subjected to integrative analysis [40]. Bioinformatics studies focused on protein interactions, annotations, and enrichment of biological processes. The investigation depicted a blunt correlation between the phosphatase and tensin (PTEN) homolog gene, along with other genes, targeting the primary regulatory responsibilities of PTEN [21].

Furthermore, the investigation portrayed the importance of genes with the methyl group in the management of resistant ovarian cancer. Such an outcome might further aid in the prognosis of ovarian cancer [21]. Moreover, in the case of off-label selection of drug amongst patients suffering from triple-negative breast cancer (TNBC), a personalized medication approach was developed, along with collation of cancer drugs, knowledge resources and drug target databases, supporting selection of targets, followed by analysis of patient information in the Cancer Genome Altar [21]. A bioinformatics-based approach was established for the selection of tumor drugs, via involving personal molecular profile data, such as mutations, copy number alterations as well as genomic expressions. The investigation recognized certain extra targets, such as protein tyrosine kinase 6 and gamma-glutamyl hydrolase, which had not been fully studied in TNBC patients [21].

The genetic expression profiles in osteoporosis were developed from information in Gene Expression Omnibus, in an investigation associated with osteoporosis-related drug targets [41]. A classical t-test method has been employed for the analysis of differentially expressed genes (DEGs). The categories of dysregulated gene ontology and dysfunctional pathways were identified by the functional pathway enrichment analysis [21]. The compounds that promoted inverse gene alterations were identified using a connectivity map. The investigation revealed enrichment of DEGs in nine pathways, including the mitogen-activated protein kinase (MAPK) signaling pathway. Additionally, sanguinarine was reported to be a potent therapeutic drug [21].

The translational bioinformatics-based strategies have been reported to be applicable for repositioning of drugs associated with organ transplantation, approved by the FDA [42]. The molecular processes were identified by applying meta-analysis of genomic information and drug databases, along with bioinformatics approaches. For instance, crucial enhancement was reported for the interleukin-17 (IL-17) process [21]. The techniques constituted analysis as well as microarray data set profiling from allograft biopsies in human kidneys, which permitted a drug repositioning technique by employing the drugs available, which would abbreviate the costs [21].

Translational bioinformatics approaches may also aid in the discovery of drugs and repositioning of existing drug candidates, in the case of inflammatory bowel disease (IBD) and other autoimmune disorders [43]. Identification of gene and microRNA biomarkers is enabled by employing bioinformatics techniques and HTP computations [21]. The drugs for repositioning can be discovered with the help of appropriate gene-level profiling of IBD subtypes, on a clinical level, and their relationship with autoimmune disorders. The IBD genes (highly expressed) might suitably target drugs for viral infections, gastrointestinal tract (GIT) cancers, and autoimmune disorders [21].

BioHCVKD is a bioinformatics tool and knowledge discovery system that was developed for the annotation and mining of suitable information, in a study, related to hepatitis C virus (HCV) [44], that collaborated with the conditional random field-based gene mention tagger and dictionary-based filtering and may aid in the identification of ligands, active residues, and proteins to potentiate the drug discovery process. A bioinformatics approach, based upon signal processing, was employed in the examination of protein residues, in the evaluation of therapies for human immune-deficiency virus/acquired immune-deficiency syndrome (HIV/AIDS) and drug resistance, by applying digital signal processing methods, such as the informational spectrum method (ISM) [45], which collated ISM, information associated with protein sequences, as well as other suitable data.

The digital technique to evaluate resistance towards a drug can be employed in other drug resistance studies to develop a computer-aided drug resistance calculator. Databases can be developed to interpret molecular associations in order to evaluate drug abuse and Neuro-AIDS [46]. Effective evaluation of gene expression interactions can aid in better understanding the depth and intensity of the problems. The potent database systems may constitute large data sets, as well as function as a database for a public domain, to facilitate queries, deposition, and review of data [21].

Numerous public databases, such as GLYCAN, Kyoto Encyclopedia of Genes and Genomes (KEGG), Consortium for Functional Glycomics, and glycoSCIENCES.de, can be employed for glycome informatics, which has been reported to aid in the analysis of data associated with the structure of the glycan [21]. Table 1 enlists the methods of translational bioinformatics for drug discovery and development.

## 4. Optimization of Anti-Microbial Research by Bioinformatics Approaches

The research in the antimicrobial paradigm has resulted in the discovery of potential anti-microbial drug candidates; however, the elevating number of antimicrobial resistance bacteria has triggered the need to develop more efficient and novel antimicrobial drug candidates. The novel genetic data can cause changes in the protein structure, which impacts the ability to carry antibiotics, enzyme-mediated inactivation of drugs, and structural alterations during interactions between bacteria and drugs [50]. Furthermore, numerous natural compounds can be used to fight against such infections, on account of their antimicrobial properties, which are referred to as antimicrobial peptides (AMPs).

**Figure 3:**
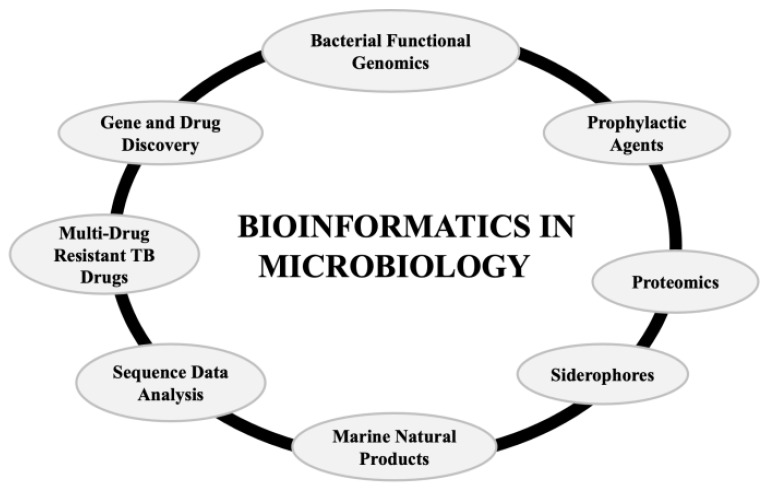
Bioinformatics in multiple assets of microbiology—proteomics, bacterial functional genomics, gene and drug discovery, siderophores, marine natural products, sequence data analysis, multi-drug-resistant tuberculosis (TB) drugs, and prophylactic agents.

Bioinformatics-associated advancements in bacterial transcriptome provide a greater understanding of varying microbial adaptations in conditions of environmental stress, which will aid in the development of novel AMPs [50]. The tools and techniques associated with bioinformatics ameliorate and short-list the total number of lead candidates to be employed as drugs and recognizes the efficient therapeutic agents. Additionally, there are numerous forms of advantages of bioinformatics in the microbiology field [50] (Figure 3).

Amongst the prime areas of bioinformatics, metagenomic shotgun sequencing (MSS) is rapidly growing, in association with mathematics, biology, and computational techniques [50]. Shotgun sequencing can be employed to attain high-resolution taxonomic composition as well as genetic profiles of metagenome samples [50]. MSS has reported limited gene richness and retarded butyrate-producing bacteria in the gut microbiome of individuals with obesity [51]. Rapid taxonomic profiling of metagenomic data is done by a bioinformatics tool, MetaPhlAn [52], by employing a database of particular marker genes of taxonomical importance, shortlisted from 3000 microbial reference genomes. The functional profiling of metagenomics information is in high demand to overcome the reduced specificity and sensitivity due to long runs in the mapping of deoxyribonucleic acid (DNA) reads and pseudohits to non-associated proteins [50].

An online service was provided by Argonne National Laboratory, namely, metagenomics rapid annotation using subsystem technology (MG-RAST), which facilitates automatic metagenomic functional profiles [53]. DNA read alignment to protein database is enabled by DIAMOND, which exhibits 20,000 times greater speed compared to the basic local alignment search tool (BLAST) × on short reads, where the sensitivity of the two is the same [54]. The protein–protein alignments are enabled by MMseq2, which is 400 times more rapid as compared to PSI-BLAST [55].

Novel approaches by employing rapid DNA–DNA alignment with Bowtie 2 [55,56,57] to the metal gear solid (MGS) database, resulting in more specific alignments. Alignment of metagenomic reads is enabled by HUMAnN2, to NCBI UniRef microbial genomes [50]. Another metagenomic tool, MGS-Fast, aids in DNA alignment, and its gene annotation is related to KEGG and IGC [50]. Multiple operational taxonomic units (OTU), which depict bacteria, which are uncultured, are developed by direct polymerase chain reaction (PCR)-amplified 16S gene sequencing [50]. About 1,719,541 16S rRNA sequences of bacteria are contained in rRNA database, SILVA, with 99% level of identity into 645,151 representative sequences [56].

Antimicrobial resistance in pathogens has triggered the development of novel antimicrobial candidates. Siderophores provided newer approaches for the establishment of suitable targets for antibiotic discovery [57]. The siderophore receptors are located on the cell membrane of the pathogen and facilitate entry of the antibiotic and produce a black hole due to deficiency of iron. Detailed study of biosynthetic pathways of siderophores permits the development of significant targets to facilitate hindering of these siderophores in pathogenic agents, which regulate pathogenic virulence, referred to as the Trojan Horse Strategy, which prevents the pathogen from becoming resistant to the drug candidate [50].

Antimicrobial resistance is accelerating from proto resistance to uncurable clinical pathogens. The effective therapy of resistant infections and discovery of novel drugs is facilitated by genotype data [50]. For instance, the estimation of resistance phenotype from genotype is enabled by the Comprehensive Antibiotic Resistance Database (CARD), which acquires curated mechanisms for resistance to data, genes as well as their targets, for resource establishment for the generation of an algorithm for the estimation of resistance to antibiotics [50]. The Resistance Gene Identifier (RGI) in the CARD presently gives an estimation of resistant genes, evaluates genome assemblies, and gives a comprehensive account of estimated genes resistant to antibiotics as well as targeted groups of drugs [50].

Furthermore, bioinformatics approaches are also employed in the development of multidrug-resistant tuberculosis (TB) drugs. The management of TB can be accelerated by using -omics technologies, where some drugs, approved by the FDA, are being validated for repurposing, followed by the development of more effective drugs with the ability to reduce tolerance towards a drug or altering the immune system response of the host [58]. The bioinformatics tools for genotyping and drug-resistant TB are listed in Table 2.

Advancements in bioinformatics and next-generation sequencing (NGS) techniques facilitate the evaluation and characterization of novel chemicals in marine natural products [59]. Bioinformatics approaches also aid in the development of prophylactic agents [50].

Bioinformatics tools are employed in the study of bacterial functional genomics. A reliable overview of the metabolic processes and phylogenetic diversity in microbial organisms is provided by integrated genome comparison systems, which comprise the prediction tools and protein functional classification systems [50]. A detailed account of the biochemical functions, metabolic processes, knockout phenotypes, inhibitors, and substrates is collated into a prioritization tool [60].

Furthermore, bioinformatics has brought about a revolution in the field of pharmacy by aiding in advancing the areas of drug discovery and target validation. This approach involves the employment of chemo-informatics in antimicrobial study, programs, machine learning approaches, fuzzy logic modeling, artificial neural networks (ANNs), genomics, and target discovery as well as molecular dynamics and simulations [50]. Numerous bioinformatics-based tools have been developed in microbiology to facilitate delivery and genes and drugs in organisms, as enlisted in Table 3.

**Figure 4:**
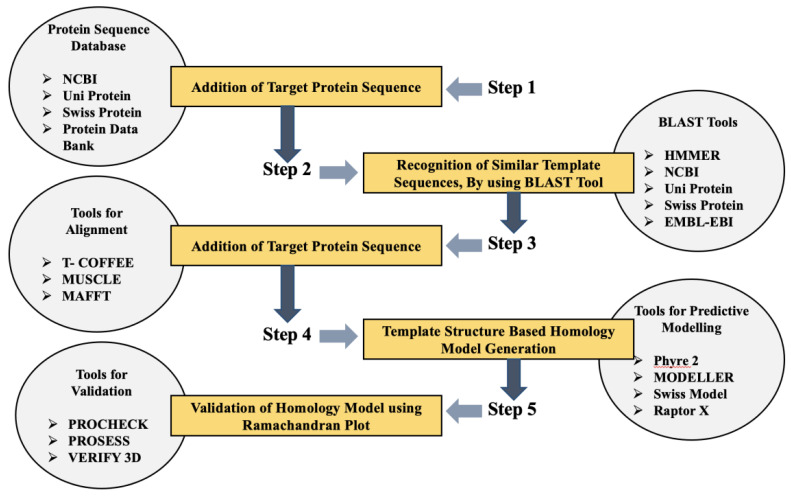
Use of bioinformatics-based tools, for alignment, validation, prediction modeling, BLAST tools, and protein sequence databases, in multiple steps of 3D homology model generation. Legend: BLAST—basic local alignment search tool; EBI—European Bioinformatics Institute; EMBL—European Molecular Biology laboratory; HMMER—biosequence analysis using profile hidden Markov models; MAFFT—Multiple Alignment using Fast Fourier Transform; MUSCLE—multiple sequence comparison by long expectation; NCBI—National Center for Biotechnology Information; Phyre 2—Protein Homology/AnalogY Recognition Engine; T-COFFEE—tree-based consistency objective function for alignment evaluation.

Furthermore, bioinformatics databases and tools have been found to aid in big data analytics, comprising the generation of a 3D homology model (Figure 3). Targeting of the NGS platform has been modified to attain diagnostic techniques at a molecular level, as a result of a proper understanding of molecular epidemiology and resistance genotype [69]. Novel data sharing prototypes have to be developed by CARD that aid in clinical research and diagnostics, using AMR computational techniques [69]. Moreover, proteomics is an accelerating field that is expected to significantly contribute to bioinformatics in the future. The computational techniques have already replaced methods such as protein microarrays, 2D gel electrophoresis, and mass spectrometry in protein structure interpretation [50]. The bioinformatics tools aid in phylogenetic profiling, metabolic pathway mapping, and expression profiling [50,70,71,72] **(**Figure 4).

## 5. An Overview of Bioinformatics in Clinical Genomic Sequencing and MicroRNA Research

The bioinformatics approaches have interlinked the biological science with computational approaches, which has proven to be revolutionary in the field of medical sciences [71]. The bioinformatics approaches have been developed for mining, regulating, and processing as well as evaluating raw information, which has been accumulated from modernized approaches, such as the next-generation sequencing technique, as well as published studies [73,74,75]. Moreover, the bioinformatics tools based upon miRNAs are further divided into primary groups, namely, miRNA and target gene, metabolic pathway of miRNA, miRNAs identification, miRNA and transcription factors, miRNA–miRNA functional network, miRNA and mutations, miRNA and diseases, etc. [74,75]. The ease of accessibility influences the significance of the database amongst the customers, whereas superior quality evaluation of data creates more favorable tools of the software [73,74,75].

### 5.1. Bioinformatics Tools in MicroRNA Research

MicroRNAs (miRNAs) are RNA entities that are non-coding, single-stranded molecules that significantly contribute to the systems regulating complex genetic expression [70]. They aid in numerous metabolic and physiological processes, namely, proliferation, evolution, apoptotic pathway, differentiation, aging, and pathological processes [71]. Multiple human genomic areas, other than chromosome Y, comprise miRNA encoding genes, either individually or as clusters [72]. Effective profiling of miRNA expression is necessary in clinical healthcare and research, as altered miRNAs contribute to multiple body disorders [73,74]. Their significance as effective biomarkers for clinical use depends upon their expression profile in body fluids. Furthermore, the techniques associated with the quantification of miRNA and collection of standardized samples are simple [75]. The development of highly effective novel techniques, such as next-generation sequencing, results in the elevation of a number of raw biodatas. Moreover, advanced integrated computational techniques, such as artificial intelligence (AI), data processing, computer science, etc., are employed in the management and evaluation of large-scale biodata [71]. The field of bioinformatics research constitutes the -omics approaches, such as transcriptomics, genomics, metabolomics, and proteomics [76]. Furthermore, numerous bioinformatics techniques are presently available for investigations related to miRNAs, as well as in silico detection of miRNA biomarkers. Different bioinformatics tools have been investigated in the microRNA field, targeting the associated pathways, mutations, targets, biomarker discovery, and diseases [71]. 

MicroRNAs have also been identified to be used as biomarkers in the detection and treatment of diseases, which has been investigated over the previous years [71]. The miRNAs have been found to play a significant role in disorders such as cardiovascular complications, neuronal disorders, autoimmune diseases, cancer, and viral diseases [77]. The unreliable treatment approaches and problems faced in accomplishing desired expression profiles in diabetes, hepatic disorders, and cancers have diverted medical attention to the significance of miRNAs as fundamental elements that are required to be studied further [71]. The proprietary bioinformatics approaches are required to facilitate the recognition of miRNAs as biomarkers [78,79]. Firstly, detailed information about a specific miRNA is provided by microRNA and intragenic data (MIRIAD) [80]. 

The tools based upon bioinformatics facilitate the evaluation of the expression pathway of biomarkers, analysis of target genes, and sequence analysis. The second step in biomarker selection constitutes recognition of cell-line and tissue-specific microRNAs, where the tissue-specific miRNA expression profile depicts bioinformatics-based tools, namely the microRNA body map and miRmine, depending upon the transcriptomic results obtained from micro-arrays pr RT-PCR techniques [71]. The tools aim to introduce the functional miRNAs that are revealed in a specific tissue [81]. With the growing significance of circulating biomarkers, blood miRNAs are considered to be effective tools in interpreting the status of biomarker agents, depending upon their cellular origin in the blood stream [82]. The ability of miRNAs as biomarkers can be evaluated by MIRUMIR, which is a tool, based upon bioinformatics, and is employed in numerous types of cancers [83]. Similarly, such tools are available for other disorders, such as cardiovascular complications, as is evident from Cardio_ncRNA database [84]. Furthermore, the triplexRNA tool facilitates monitoring of 2D interaction between target genes and biomarkers [85]. Similarly, miRNA-drug and miRNA-virus interactions are determined by bioinformatics tools such as the VIRBase and mammalian transcriptomic database (mTD), which facilitates the selection of biomarkers of therapeutic importance [86,87].

Different approaches based upon bioinformatics offer multiple facilities, which have been further elaborated. The manuscript targets the text mining and meta-databases, based upon bioinformatics, such as OMIC tools [88] and Tools4miRs [89], which were employed to attain numerous bioinformatics tools associated with microRNAs. Different bioinformatics tools belonging to multiple categories have been evaluated, as listed in Table 4 below.

### 5.2. Bioinformatics Process in Clinical Genomic Sequencing 

The molecular genetic pathological paradigm has been transformed from a visual approach to an informatics-dependent strategy with the advent of high-throughput sequencing technologies. The advantages, such as enhanced efficiency, ameliorated costs of sequencers, data storage, and data computation, have permitted access to large-scale sequencing (genomes and exomes) to a broad range of patients [115]. These consist of deep evaluation of tumor-normal pairs, detection and screening of rare genetic maladies, and diagnosis of healthy subjects. The elevating amount of information has enhanced the significance of the field of clinical bioinformatics [115]. Therefore, it is necessary to understand the importance of clinical bioinformatics in the present molecular genetic workflow pattern, where this section aims to provide a general outlook process, required to put clinical bioinformatics into action in the field of genomic sequencing. Informatics is fundamental in numerous spheres of laboratory testing, but this section accounts for computational approaches, parsing, and interpretation from sequencing tools, via a set of variants [115]. 

**Figure 5:**
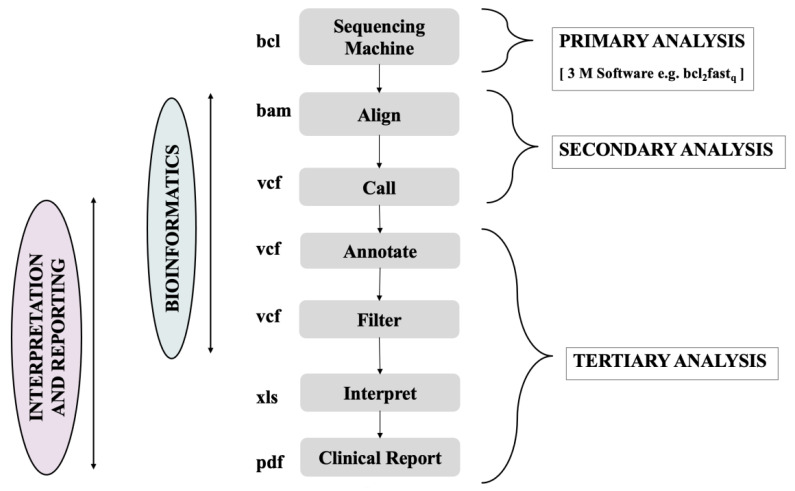
Role of bioinformatics in primary, secondary, and tertiary analysis in clinical genome sequencing, employing tools in sequence machine, alignment, calling, annotation, filtering, interpretation, and preparation of clinical reports.

Figure 4 portrays a typical bioinformatics process in clinical genomic sequencing where the whole pathway is segregated into the primary, secondary, and tertiary interpretation. The algorithms related to the sequencing tools, which deal with transforming raw sequence reads into a string of arsenic (As) and cesium (Cs), constitute the primary analysis [115]. The alignment or mapping of the sequence reads on the reference genomic sequence is detailed in the secondary analysis, followed by variant calling or recognition of variations between the reference genome and individual’s sequence [115]. All the steps that are required to analyze the recognized sequence variants are included in tertiary analysis, which comprises filtering and annotating the recognized variants in order to find variations that are clinically relevant. The steps or phases of the process of clinical bioinformatics are followed by the quality control process, which portrays the robustness, completeness, and reliability of the generated data [115] (Figure 5).

Genome-scale sequencing is consistently advancing in both detection and screening of healthy individuals as well as newborn babies [115]. More rapid genome sequencing will be enabled, with greater accuracy, on account of multiple advancements in technological parameters, algorithms, and computation, which include [115]:

- A reference genome based upon graphical elucidation, which will promote a complete genome pathway with greater accuracy in alignment, especially for ethnicities that are under-represented.
- A longer read sequencing, which will promote greater genome resolution with greater accuracy in alignment.
- A software based upon a graphics processing unit (GPU), which will promote elevated parallelization and rapid computation.

Genomic sequences have begun to utilize basic computational advances, especially around big data. The accuracy of variant interpretations has been reported to be elevated by using natural language processing and machine learning, which is also employed in variant calling [116]. Full genome interpretation will be enabled by data integration, which will not be limited to the coding areas that are the prime target of the present clinical interpretation workflows. In the combined form, these technological tools will help to expose the potential of the genetic sequence of an individual within the area of clinical practice [115] (Table 5).

## 6. The COVID-19 Pandemic Giving an Impulse to the Bioinformatics Approaches

The severe acute respiratory syndrome coronavirus 2 (SARS-CoV-2) is responsible for the coronavirus disease 2019 (COVID-19), which has put a tremendous amount of pressure on the global healthcare system and has taken millions of lives across the globe [140]. Multiple novel vaccines have been developed by different areas of the healthcare sector, constituting medicine, public health, biology, computer science researchers, and bioinformatics [141]. Multiple themes are associated with COVID-19 and SARS-CoV-2 research, from a biological perspective, comprising HTP technologies such as Next-generation sequencing for detection of SARS-CoV-2 genome, databases for storage of SARS-CoV-2 variants and genomes, and databases and software tools based upon bioinformatics approaches for the evaluation and investigation of interactions between host and virus [142].

In particular with the therapeutic interventions, the major research themes comprise the identification of biomarkers associated with COVID-19, discovery of drug targets, and bioinformatics methods for drug repurposing, which is employing already-developed and available drugs for COVID-19 management [143]. The primary research themes at the public health and epidemiological level comprising ordered assemblage and release of information about the infection spread, such as regular reports of number of cases, number of deaths, number of patients hospitalized and in ICU, etc., may aid the general population to realize the severity of the infection [144], followed by biological tests for testing and evaluation and computational tools and techniques to track the infected individuals, storage of large amount of clinical data in electronic health records of infected patients [145], evaluation and assessment of the impact and effects of complete lockdown at a socioeconomic level, and techniques to aid quarantined people by using local services, virtual assistants, and advanced technologies such as robots [143].

Complex techniques are used in the case of viruses to potentiate the coding ability of genomes due to the small size of the genome [146]. Bioinformatics, in collaboration with genomics, has provided a significant amount of information to understand the pathogenetic mechanisms as well as the spread of anti-microbial resistance to the immune responses of host cells in infectious diseases [147]. The size of the genetic sequence of novel severe acute respiratory syndrome 2 (SARS-CoV-2) has been reported to vary from 29.8 to 29.9 kb, with sequence variation as compared to the human coronaviruses, such as SARS and Middle East Respiratory Syndrome (MERS), recognized earlier [148]. However, it is essential to investigate the virological, epidemiological, and pathogenic data of SARS-CoV-2 in order to evaluate newer therapies and to promote the development of efficacious strategies to prevent the spread of the disease [149,150,151]. Bioinformatics strategies and tools have been utilized in the investigation of SARS-CoV-2 in order to minimize and control the negative impact of this pandemic, which has been observed to deteriorate the health of the population as well as the global socio-economic status [142].

### 6.1. Bioinformatics Tools and Techniques for COVID-19 Research

As a result of significant research in this regard, many scientific publications have been made available, along with novel tools and techniques to study the COVID-19 literature, such as data mining as well as natural language processing, to extract appropriate information [6]. The genomic data of SARS-CoV-2 can be assessed and detected by using next-generation sequencing that gives access to general information about the virus, followed by computation of the data and extending the knowledge by using bioinformatics pipelines as well as biological databases related to the interactions between host cell and virus. 

Bioinformatics tools have also been developed for the detection, evaluation, and treatment of the disease exhibition fundamental actions such as SARS-CoV-2 detection, sequencing data analysis, tracing and containing the spread of the pandemic, the study of the evolution of the coronavirus, effective drug target discovery, and therapeutic approaches [143]. Data integration has been reported to aid in the assessment and identification of SARS-CoV-2 genome sequences and metadata, along with host–pathogen integrated datasets and integrative surveillance mechanisms [143]. Furthermore, the role of pathway enrichment analysis (PEA) in identifying suitable viral targets in biological processes in the host has also been reported. The online resources and Web tools focus on significant parameters of the virus, comprising genomics, interactomics, epidemiology, and pharmacology [143].

The COVID-19 pandemic has also provided an impulse for drug repurposing and 3D modeling, where the former deals with discovering the applications and roles of already-available drugs, resulting in amelioration of cost and time consumption, and the latter comprises oligomer and protomer models, SARS-CoV-2 human protein interactions, protein-ligand docking, and the effect of mutations [143]. This section primarily focuses on three bioinformatics approaches, next-generation sequencing (NGS), genome-wide association study (GWAS), and computer-aided drug design (CADD), as discussed further. Table 6 highlights multiple tools and databases, based upon bioinformatics, that can be employed in the management of the current pandemic situation.

#### 6.1.1. Next-Generation Sequencing (NGS)

The advancement NGS has optimized the intensity and scale of biomedical sciences. In the healthcare paradigm, during the condition of an outbreak or a pandemic, it is essential to effectively and rapidly identify the causative agent/pathogen responsible along with epidemiological surveys and analysis, which are essential to promote disease control reaction [142]. Furthermore, whole-genome sequencing (WGS), metagenomics strategy, and other high-throughput sequencing techniques promote the opportunity to obtain the full sequence of the disease-causing genome [142]. Metagenomics is a simple and economical technique that is independent of the reference sequence for analysis, unlike the in silico virus sequencing [142]. It has been identified as a powerful tool for the identification of pathogens from environmental samples, followed by direct genomic evaluation of the organism during the conditions of pandemics or outbreaks [179]. 

Similarly, in the current COVID-19 pandemic, metagenomics has been applied to provide significant novel data related to SARS-CoV-2, along with rapid recognition and characterization of the initial few COVID-19 cases [180,181], to promote the examination of SARS-CoV-2 parallel to the other co-infections in nazo-pharyngeal throat swab samples of patients [182], the intermediate recognition of the host responsible for infection transference to the human body [183], SARS-CoV-2 homologous sequence screening in other organisms [184], the impact of SARS-CoV-2 on changes in fecal microbiota in humans [185], the clinical infection of SARS-CoV-2 along with co-infections of bacteria [179], and so on. Such observations aid the clinicians and researchers to understand and isolate the COVID-19 patients, depicting different symptoms in a better way. 

The applications of metagenomic strategies can be interpreted by numerous databases and software [142]. The limited load of the viral genome, unlike the host DNA, and problems associated with accurate genome assembly pose a significant challenge to directly obtaining the genome sequence of the virus from clinical samples [142]. The study of evolution and the genetic relationship of the virus to diseases as well as tracking the outbreaks is enabled by the WGS technique, which is considered an efficient strategy on account of the intensity of the sequencing data and quality of the sequences obtained [186]. 

Samples were collected from various countries globally by employing NGS techniques such as Roche, Illumina MiSeq, etc., and WGS of SARS-CoV-2 was carried out in order to facilitate early diagnosis and interpretation of COVID-19 disease [187,188,189]. Nanopore sequencing was employed for genetic material sequencing of SARS-CoV-2 [189]. The whole-genome sequence, available on different online databases as well as data analysis software, has been reported to optimize the genomic data analysis and offers the administration of better medications to the patients [142]. 

#### 6.1.2. Genome-Wide Association Study (GWAS) 

The GWAS has established a significant relationship between complex characteristics of humans and disorders. Translational genomic research requires effective and comprehensive identification of variants from WGS [190]. GWAS is associated with the detection of variants across the genomic sequences of various individuals to detect the relationship between genotype and phenotype [142]. These genetic variants identified are employed in the recognition of individuals susceptible to deadly disorders, which affects early screening and prevention of illness [191]. GWAS is a potential genetic analytical technique of observable alleles related to the disorder in the host cell, as single nucleotide polymorphisms (SNPs) [192]. GWAS is associated with certain applications, such as genetic or nucleotide alterations in the form of SNPs, sequence analysis, alignment, structural changes in the genomic sequence, primer design, and so on, which have developed newer approaches in SARS-CoV-2 investigations by effectively identifying as well as quantifying rare viral variants in the species [148,193]. The haplotype diversity analysis has been incorporated along with phylogenetic analysis in research analysis, associated with SARS-CoV-2, in order to investigate the population demography and evolution of SARS-CoV-2 across the globe [194,195]. The phylogenetic study has been reported to be applicable to the investigation of evolutionary and molecular association of SARS-CoV-2 with other coronavirus species, which further provided necessary information for efficient evaluation of genomic sequence of SARS-CoV-2 [193,196,197,198].

Certain primers are developed via in silico algorithms by focusing on conserved segments in the viral genetic material in order to ameliorate false-positive results during COVID-19 testing via a real-time polymerase chain reaction (rtPCR) as well as retard the requirement for standardization across varying PCR protocols [199,200]. These data related to infectious genes of SARS-CoV-2 are aiding the researchers across the globe in the generation of a vaccine to combat the virus, as per the detected virus genes coding regions, molecular alterations, and genetic sequence variations between the isolated species globally [142]. The genomic analysis and experimental investigations comprising phylogenetic analysis, single nucleotide polymorphism (SNP) study, primer designing, and so on have been conducted via high-throughput bioinformatics strategies and technologies, which are reported to be effective in data annotation and analysis [142]. 

#### 6.1.3. Computer-Aided Drug Design (CADD) 

The drug design process is considered to be a challenging approach that is cost-ineffective as well as time-consuming [201]. The drug design is incorporated with bioinformatics, which has become a significant part of this process and plays an essential role in drug target validation. It aids in understanding the complexity of biological pathways in order to enhance the drug discovery process [202]. CADD has been reported as a dominant process on account of its appropriate algorithms, comprising digital repositories’ establishment, for the investigation of associations of chemical interactions [142]. 

Computational techniques for designing compounds with unusual physico-chemical properties along with assessment tools for the evaluation of potential lead candidates and so on aid in the discovery and development of drugs [203]. This method is associated with further advantages, such as cost-effectiveness, insight knowledge of interaction between drug and receptor, time to market, and an accelerated drug discovery and development process, which elevates its popularity in the research field associated with science [194]. 

## 7. Future Prospects and Conclusions 

The bioinformatics assets can be modified to further improve the diagnostic and detection criteria and procedures in the healthcare sector. The employment of bioinformatics tools in the growing fields of pharmacovigilance and genomic sequencing holds great future benefits. Future transformations in the techniques and tools of bioinformatics can aid in better understanding drug resistance and microbial virulence, which can facilitate effective management of viral infections. There are numerous health disorders for which proper and reliable treatments are not yet available, such as cancer, HIV-AIDS, neurodegenerative diseases, etc. There is an absence of a significant research model in pharmacovigilance that would aid in providing a focused direction and scope of this field in the future, resulting in potential benefits [204]. The bioinformatics-based computational techniques can facilitate the acceleration of the drug development criteria, which can further result in the development of more active therapeutic candidates with limited toxicity profiles. Furthermore, the decision-making tools and comprehensive models can aid in transforming the conventional processes of drug delivery from single target to ‘function first’ as well as phenotypic selection methods, targeting systematic networks [21]. Taking into account the pandemic situation, the researchers can investigate and evaluate the SNPs associated with the affected host body, and computational primer design algorithms can be used to design modified forms of newer primers of genes or nucleotides [142]. Therefore, the bioinformatics approaches would facilitate simulation, identification, and prediction of the progression of the disease and responses of the drug candidates, for elevating the uses, safety profiles, and impact of newer and existing drugs, thereby strengthening the healthcare system. 

Bioinformatics has accelerated the field of biomedical sciences and has potentiated the clinical and general aspects of the healthcare system. The review focuses on giving a detailed account of the significance of bioinformatics in the pharmaceutical industry and pharmacovigilance, followed by the fundamental assets of bioinformatics-based tools and databases in drug discovery and development. The value of translational bioinformatics approaches intensifies the development and discovery of suitable drug candidates, where the manuscript enlists numerous databases in tabular forms. Moreover, bioinformatics has been reported to be employed to accelerate microRNA research and clinical genomic sequencing, where it is assessed for its potential in -omic technologies and studies. The role of bioinformatics approaches in microbiology is explained owing to its significance in gene and drug discovery, proteomics, sequence data analysis, bacterial functional genomics, and the development of multi-drug resistant TB drugs, prophylactic agents, marine natural products, siderophores, and so on. In addition, the authors reveal the positive outcomes of this tech-driven strategy in the management of the current COVID-19 pandemic, where its involvement in next-generation sequencing, genome-wide association study, and computer-aided drug design tends to strengthen the fight against the pandemic. In addition, the review aims to provide a clarified image of bioinformatics and invokes the necessity of technological tools, databases, and software, thereby attracting the readers and researchers to exhibit future assessment of this field to facilitate acceleration and elevated efficiency of the healthcare system.

## Figures and Tables

**Table 1:** Techniques of translational bioinformatics for discovery and development of drugs.

Diseased States	Translational Bioinformatics Techniques	Refs.
Osteoporosis drug targets	Functional pathway enrichment, genetic expression profiles from GEO, dysfunctional pathways	[41]
Neuro AIDS and drug abuse	A public domain database, molecular relationship-evaluating database	[46]
Repositioning of drugs	Evaluation of transcriptomic information for relationship between drugs and disease	[47]
Drug resistance in ovarian cancer therapy	Evaluation of protein interactions, evaluation of methylated genes, related to drug resistance, enrichment of biological process	[40]
Drugs for AIDS and drug resistance	Calculator of resistance to drugs, evaluation of residues of protein with digital signaling processing	[45]
Repositioning of drugs in transplantation of organs	Microarray dataset profiling, meta-analysis of genomic drugs as well as information, recognizing redundant molecular processes	[42]
HCV drug discovery	Collation of filtering, based upon dictionary, and gene mention tagger, knowledge discovery process for literature mining	[44]
Off-label selection of drugs for TNBC	Evaluation of TNBC patient information, collaboration of databases of cancer drugs and respective targets, evaluation of personal molecular profiles	[48]
Glycomics and drug targets	Tree-based algorithmic models for glycan structure data, collation of data, public databases for glycome informatics, such as KEGG	[49]

Legend: AIDS—acquired immune-deficiency syndrome; GEO—gene expression omnibus; HCV—Hepatitis C virus; KEGG—Kyoto Encyclopedia of Genes and Genomes; TNBC—triple-negative breast cancer.

**Table 2:** Bioinformatics tools for genotyping and drug-resistant TB.

Bioinformatics Tools	Type	Genotyping
PhyTB	Online	SNP
CASTB	Online	4a
TGS-TB	Online	4b
KvarQ	Stand-alone	SNP/Spol
TB-Profiler	Online	SNP
PhyResSE	Online	SNP

Legend: PhyTB—phylogenetic tree visualization and sample positioning for *Mycobacterium tuberculosis*; CASTB—comprehensive analysis server for the complex *M. tuberculosis*; TGS-TB—Total Genotyping Solution for *M. tuberculosis;* KvarQ—tool that directly scans fastq files of bacterial genome sequences for known variants; TB-Profiler—Profiling tool for *M. tuberculosis* to detect drug resistance and lineage from Whole-Genome Sequencing Data; PhyResSE—a Web Tool Delineating *M. tuberculosis* Antibiotic Resistance and Lineage from Whole-Genome Sequencing Data; SNP—single nucleotide polymorphisms; Spol—database.

**Table 3:** Bioinformatics tools in microbiological paradigm of gene and drug delivery.

Bioinformatics Tools	Role	Refs.
AntiBP server	Anti-bacterial peptides prediction in a protein sequence	[61]
BACTIBASE incorporated with MODELER	Prediction of 3D structure of user peptide by homology to known bacteriocins; such relational database is applicable for in silico designing of newer AMPs.	[50]
Titanium	Capable of making 400–600 million bases/run with 400 base pairs’ read lengths	[50]
454 sequencing technology	Employed in studies related to 16S profile of microbiomes	[62]
Illumina sequencing platform	Rapid outputs due to more accurate outcomes and read lengths	[50]
MiSeq and HiSeq	Production of sequencing data of 100 GB in just 6 days	[63]
ANNs	Mathematical modeling algorithms, which provide an effective and reliable method for in silico detection of newer AMPs	[64]
Fuzzy logic modeling	Accurate evaluation of AMPs, enables antimicrobial research and development in quantitative structure–activity relationship (QSAR)	[65]
Machine learning	Evaluation of high-dimensional gene expression datasets for selection of gene	[66]
CHARMM	Program employed to facilitate activation of AMP interactions with lipid bilayers	[67]
GROMACS	Freeware tool employed in MD studies to develop trajectories	[68]

Legend: AMPs—antimicrobial peptides; ANNs—artificial neural networks; AntiBP—anti-bacterial peptides; BACTIBASE—database dedicated to bacteriocins; CHARMM—biomolecular simulation program; GB—gigabyte; GROMACS—Groningen machine for chemical simulations; MD—medical doctor; MiSeq and HiSeq—sequencing technologies developed by Illumina; QSAR—quantitative structure-activity relationship.

**Table 4:** Bioinformatics-based databases and software for discovery and evaluation of miRNAs.

Tool	Category	Significance	Refs.
DIANAmicroT-CDS	Predicted miRNA target evaluation	A web-based application that facilitates data interpretation, evaluates functions exhibited by ncRNAs in body processes and diseases, and scrutinizes expression regulation datasets and miRNA regulatory elements.	[90]
MiRBase	Search for miRNAs	Introduces miRNA-based novel genes, provides comprehensive data on immature and mature miRNAs, provides immediate access to all the miRNA-related published data and resources	[91]
MiRDB	Predicted miRNA target evaluation	Predicts and evaluates the role of target genes, offers comprehensive data, provides screening options to facilitate role-based anticipation of different miRNAs, facilitates alignment of sequences	[92]
MiRscan	Search for miRNAs	A web-based application that recognizes and contrasts miRNA genes in the genetic sequence of greater than one organism	[93]
microTar	Predicted miRNA target evaluation	A windows application that evaluates the effect of miRNA binding on the whole mRNA molecule	[94]
MiReader	Search for miRNAs	A Linux and Windows application that recognizes the sequences of mature miRNAs without the requirement for reference genome sequences	[95]
miRmap	Predicted miRNA target evaluation	A windows/web-based application that is a mixture of characteristics from PITA, TargetScan, PACMIT, and miRanda, offers user-friendly approaches for operating precomputed predictions and modeling of miRNA targets	[96]
MiRanalyzer	Search for miRNAs	A windows/web-based application that is an sRNA toolbox, which recognizes or evaluates miRNA features based upon the outcome of the next-generation sequencing approaches	[97]
MiRNAPath	miRNAs and metabolic pathway	Depicts the inter-association between gene, miRNA, and metabolic pathway inputs, thus used to study the miRNA-based metabolic pathways	[98]
MiRmaid	Search for miRNAs	A web-based application that comprises all the characteristics of the miRNA database	[99]
MiRecords	Assessment of confirmed and estimated targets	Search and review of targets for miRNAs	[100]
Pharmaco-miR	MiRNAs, genes, and drugs	Significant in pharmacogenomics; integrates function status to the expression profile of miRNA via validated experimental proofs and computational techniques	[101]
MiRwalk	Assessment of confirmed and estimated targets	Validation of newer MiRNA targets, comprehensive database	[102]
ViTa	Viruses and miRNAs	Curates the target sites of miRNAs in chicken, mice, humans, or rats, with miRBase-derived known viral miRNA genes	[103]
TMREC	MiRNA regulatory network	Evaluation of role of regulatory processes, controlled by interactions between transcription factors and miRNAs in diseased states	[104]
MiR2GO	Mutations and miRNAs	Evaluation of impact of mutations and alterations in single nucleotide in central core miRNA sequence, coupling with target mRNA on their function; comparison and assessment of similarity percentage of miRNA pair functions at a cellular and molecular level	[105]
DAVID	MiRNA regulatory network	Effective interpretation of changes in a large number of genes; evaluation of procedure of functional product generation and genetic expression	[106]
PolymiRTS	Mutations and miRNAs	Evaluation of genetic polymorphisms in central core binding or target pairing site	[107]
PhenomiR	Diseases and miRNAs	Relationship between miRNA and disease	[108]
MiREnvironment	Environment and miRNAs	Interaction between environmental factors and miRNAs	[71]
MiRcancer	Diseases and miRNAs	Relationship between miRNA and cancer	[109]
CircuitsDB	Transcription factors and miRNAs	Interaction between miRNA and transcription factors to facilitate regulation of joint target gene	[110]
MiR2disease	Diseases and miRNAs	Portrays validated and estimated gene targets upon miRNA changes in diseases	[111]
PutmiR	Transcription factors and miRNAs	Interaction between miRNA and transcription factors to facilitate regulation of gene expression	[112]
MiRgator	MiRNA-miRNA interaction	Deep sequencing miRNA database that facilitates massive data analysis, comprehensive evaluation of target genes, expression profiles of miRNA–miRNA interactions, proper representation of miRNAs genes chromosomal region	[113]
DIANA-mirExTra	MiRNA-miRNA interaction	Functional evaluation of expression profiles and targets of miRNAs	[114]

Legend: Bioinformatics-based databases and software for discovery and evaluation of miRNAs. Significance of tools targeting the following categories: 1. Predicted miRNA target evaluation, 2. miRNA search, 3. miRNA metabolic pathway, 4. Confirmed and estimated targets assessment, 5. miRNAs, genes, and drugs, 6. Viruses and miRNAs, 7. miRNA regulatory network, 8. Mutations and miRNAs, 9. Disease and miRNAs, 10. Environment and miRNAs, 11. Transcription factors and miRNAs, 12. miRNA-miRNA interaction. DIANA—displacement analyzer; MiR—micro ribo-nucleic acid; MiRDB—miRNA database; MicroTAR—miRNA target prediction program; miRNAPath—miRNA and metabolic pathway; Pharmaco-miR—Pharmacogenomics and miRNA; ViTa—visual interpretations with three-dimensional annotations; TMREC—database for transcription factor and miRNA regulatory cascades in human diseases; miR2GO—comparative functional analysis for microRNAs; DAVID—the database for annotation, visualization, and integrated discovery; PolymiRTs—polymorphisms in microRNA target site; Phenotypic analysis of miRNA; PutmiR—putative transcription factor and micro RNA.

**Table 5:** Softwares used for secondary analysis and variant annotation in clinical genomic sequencing.

Name	Application	Refs.
**For secondary analysis**		
GATK-UnifiedGenotyper	Consistently proceeds towards HalotypeCaller	[117]
BWA-SW	Shorter reads alignment	[118]
Freebayes	Based upon Bayesian haplotype, efficient in evaluating genomes with specific properties	[119]
Novoalign	Alignment software for commercial use	[120]
BCF tools and VCF tools	Distinct analytical characteristics, yet some common functions	[119,121]
BWA-MEM	Longer reads alignment, with bp > 100	[118,122]
FastQC	Evaluation of sequencing quality	[123]
Picard Tools	Provides QC evaluation for multiple secondary analysis stages	[124]
GenomeStrip	Evaluation of read length, read depth, and read mate pairing	[125]
verifybamID	Detection of sample contamination	[126]
BreakDancer	SV detection	[127]
Vcfeval	Comparison of two distinct variant call files, significant for validation	[128]
Pindel	Detection of large deletions and limited insertions	[121]
Bedtools	Manipulation of multiple bed files	[129]
Manta	Germline and somatic evaluation	[130]
VisCap	CNV calling for panel information	[131]
XHMM	CNV calling for exome information	[132]
2. **For variant annotation**		
VEP	Annotation and prediction of impact of variant on genes	[133]
WGSA	Integration of results from SnpEff, VEP, and ANNOVAR, for annotation based upon gene modeling, integration of numerous epigenomics projects, integration of conservation scores, database associated with the disease, multiple prediction scores, and allele frequencies for SNV-centric resources	[134]
DANN	Integration of multiple annotations into one metric, annotation of non-coding and coding variants	[135]
CADD	Integration of multiple annotations into one metric, annotation of non-coding and coding variants	[136]
ANNOVAR	Annotation based upon gene, region, and filter	[137]
Oncotator	Aggregation of annotations from genomic, cancer variants, protein, and non-cancer variant annotations	[138]
SnpEff	Annotation and prediction of the impact of variants on genes	[139]

Legend: Software used in the process of clinical genomic sequencing, targeting secondary analysis (detailing the alignment or mapping of the sequence reads on the reference genomic sequence) and variant annotation (deals with assigning data to DNA variants). GATK—genome analysis tool kit; BWA-SW—Burrows–Wheeler aligner software; BCF—binary variant call format; VCF—variant call format; FastQC—fast quality control software; XHMM—eXome-Hidden Markov Model; VEP—variant effect predictor; WGSA—whole-genome sequencing annotator; DANN—deleterious annotation of genetic variants using neural networks; CADD—computer-aided drug design; ANNOVAR—annotate variation; SnpEff—single nucleotide polymorphism annotator.

**Table 6:** Bioinformatics-based tools and databases with the potential to combat the COVID-19 pandemic.

Bioinformatics-Based Tools/Databases	Role	Refs.
SRA database	High-throughput sequencing data repository	[152]
Fast QC	Quality control check on raw sequences in WGS	[153]
AUGUSTUS	Gene prediction in eukaryotic genome sequencing	[154]
MaSuRCA	Assembly of genome	[155]
Prokka	Prokaryotic genome annotation in WGS	[156]
Cutadapt	Recognizing and eliminating adaptor sequences, poly A tails, primer, and other unrequired sequences in WGS and metagenomics	[157]
Ragout	Reference-assisted assembly tool in WGS	[158]
Gene expression omnibus (GEO) database	Repository of data related to functional genomics	[159]
dbSNP	Repository for single-base nucleotide substitutions in SNP discovery	[160]
UCSC genome browser	Collection and analysis of model organism annotations in genomics	[161]
PROVEAN	Estimations of effect of substitution of amino acid on biological role of protein in SNP discovery	[162]
Kyoto encyclopaedia of genes and genome (KEGG)	Analysis of metabolic pathway	[163]
SIFT	Estimation of amino acid substitution on functional role of proteins	[164]
Conserved domain (CD) Search	Sequence alignment	[165]
NCBI gene database	Genetic data repository	[166]
PAUP	Evaluation of phylogenetic relationship between molecular sequences by using parsimony method	[167]
UniProt	Stores functional data on proteins	[168]
PopArt	Phylogenetic evaluation with visualization of haplotype diversity network	[169]
Molecular evolutionary genetics analysis (MEGA)	Alignment of multiple sequences, generation and statistical evaluation of phylogenetic relationships	[170]
Primer 3	Primer design in high-throughput genomics	[171]
PubChem	Chemical structure database for drug designing	[172]
Basic local alignment search tool (BLAST)	Finding similarity between sequences	[173]
AutoDock, Patch dock, Swiss dock, Zdock	Molecular docking tools	[142]
Protein databank (PDB)	3D-protein structure database	[174]
Drug bank	Comprises of data on FDA approved drugs	[142]
Modeler	Homology of 3D protein structures	[175]
PyMol	Editing and visualization of molecular structure	[176]
GROMACS	Tool for simulation of molecular dynamics	[142]
NAMD	Parallel molecular dynamics code	[142]
Open Babel	Chemical toolbox aiding in drug designing	[177]
VMD	Built-in scripting and 3D graphics-based visualization program	[178]

Legend: Bioinformatics-based tools and databases with the potential to combat the COVID-19 pandemic. Techniques and databases associated with high-throughput sequencing, whole-genome sequencing, data repositories, drug designing, molecular docking, phylogenetic evaluation, metabolic pathway analysis, sequence alignment, 3D homology, simulation of molecular dynamics, etc. SRA—sequence read archive; Fast QC—fast quality control; Ragout—Reference-assisted genome ordering utility; Prokka—prokaryotic genome annotation; Cutadapt—cutting adaptor sequences; GEO—gene expression omnibus; dpSNP—single nucleotide polymorphism database; UCSC Genome Browser—The University of California Santa Cruz Genome Browser; PROVEAN—protein variant effect analyzer; KEGG—Kyoto encyclopedia of genes and genome; SIFT—sorting intolerant from tolerant; CD—conserved domain; NCBI—National Centre for Biotechnology Information; PAUP—phylogenetic analysis using parsimony; UniProt—Universal Protein resource; PopArt—population analysis with reticulate tress; MEGA—Molecular evolutionary genetics analysis; BLAST—basic local alignment search tool; PDB—protein databank; PyMol—Python using molecular graphics tool; GROMACS—Groningen machine for chemical simulations; NAMD—nanoscale molecular dynamics; VMD—visual molecular dynamics.
